# Uterine Tumor Resembling Sex-Cord Tumor: A Case Report

**DOI:** 10.7759/cureus.12010

**Published:** 2020-12-10

**Authors:** Azhar A Sh. Hassan, Anfal A Alsultan, Raghad K Al Ghamdi, Naif M Albluwi, Jawad H Aljamea, Hassan M AlHammadi, Mujtaba J Alzakari, Mahdi H Almisbah, Fatima A Alsubaie, Modhi S Alajmi

**Affiliations:** 1 Family and Community Medicine, Imam Abdulrahman Bin Faisal University, Dammam, SAU

**Keywords:** uterine neoplasm, abnormal uterine bleeding, sex-cord tumors

## Abstract

Uterine tumors resembling sex-cord tumors are a rare group of tumors with uncertain etiology and histogenesis. The sex-cord tumors are classified into two groups. The first group includes endometrial stromal tumors with foci of sex cord differentiation less than 50% while the second group is composed predominantly or exclusively by sex cord-like elements. We report the case of a middle-aged woman who presented with heavy vaginal bleeding with initial ultrasound findings suggestive of uterine leiomyoma. There was no improvement noticed after a trial of medical treatment; hence, the surgical treatment in the form of total abdominal hysterectomy was undertaken. A few weeks after the surgery, the patient presented with unexplained abdominal pain. Imaging studies demonstrated a hypermetabolic lesion in the upper part of the vagina that was suspicious for malignancy. Complete resection of the mass was performed along with para-aortic lymphadenopathy. Histopathological examination revealed a uterine tumor resembling a sex-cord tumor. Uterine tumors resembling sex-cord tumors are a unique group of uterine neoplasms that exhibits diverge clinical and biological characteristic. Surgical pathologists must recognize this rare entity and differentiate it from other lesions.

## Introduction

Uterine tumors resembling sex-cord tumors are a rare group of tumors accounting for less than 0.5% of all uterine malignancies [1]. These tumors have uncertain etiology and histogenesis. They are often seen in premenopausal or postmenopausal women [2]. The first case of these tumors was reported in 1945 by Morehead and Bowman [3] as they described a case of uterine tumor resembling a granulosa cell tumor. The concept of sex-cord differentiation of uterine tumors was clarified by Clement and Scully as they classified the tumors into two groups [4]. The first group includes endometrial stromal tumors with foci of sex cord differentiation less than 50% while the second group is composed predominantly or exclusively by sex cord-like elements [5]. The distinction between these groups is very important as the first group has a tendency for invasion. Herein, we report the case of a middle-aged woman with a complaint of heavy menstrual periods who was found to have uterine tumors resembling sex-cord tumor.

## Case presentation

A 41-year-old woman presented to our gynecology clinic with a long history of heavy menstrual bleeding. The bleeding was associated with abdominal discomfort. There was no history of bleeding from any other sites. She did not complain of generalized fatigue, anorexia, or weight loss. Her medical history is remarkable for diabetes mellitus that is well-controlled with anti-diabetic agents. The family history was non-contributory.

The patient reported that she had not undergone an investigation for her condition. She was offered medical treatment in the past, but it did not result in any improvement. The results of coagulopathy screening were normal. Transabdominal ultrasonography revealed a hypoechoic uterine lesion suggestive of uterine leiomyoma. In light of the patient’s age and prior unsuccessful medical therapy, she was offered a surgical treatment in the form of a total abdominal hysterectomy. It is worth-noting that the quality of life of the patient was significantly affected as she needed frequent blood transfusions because of her heavy periods.

**Figure 1:**
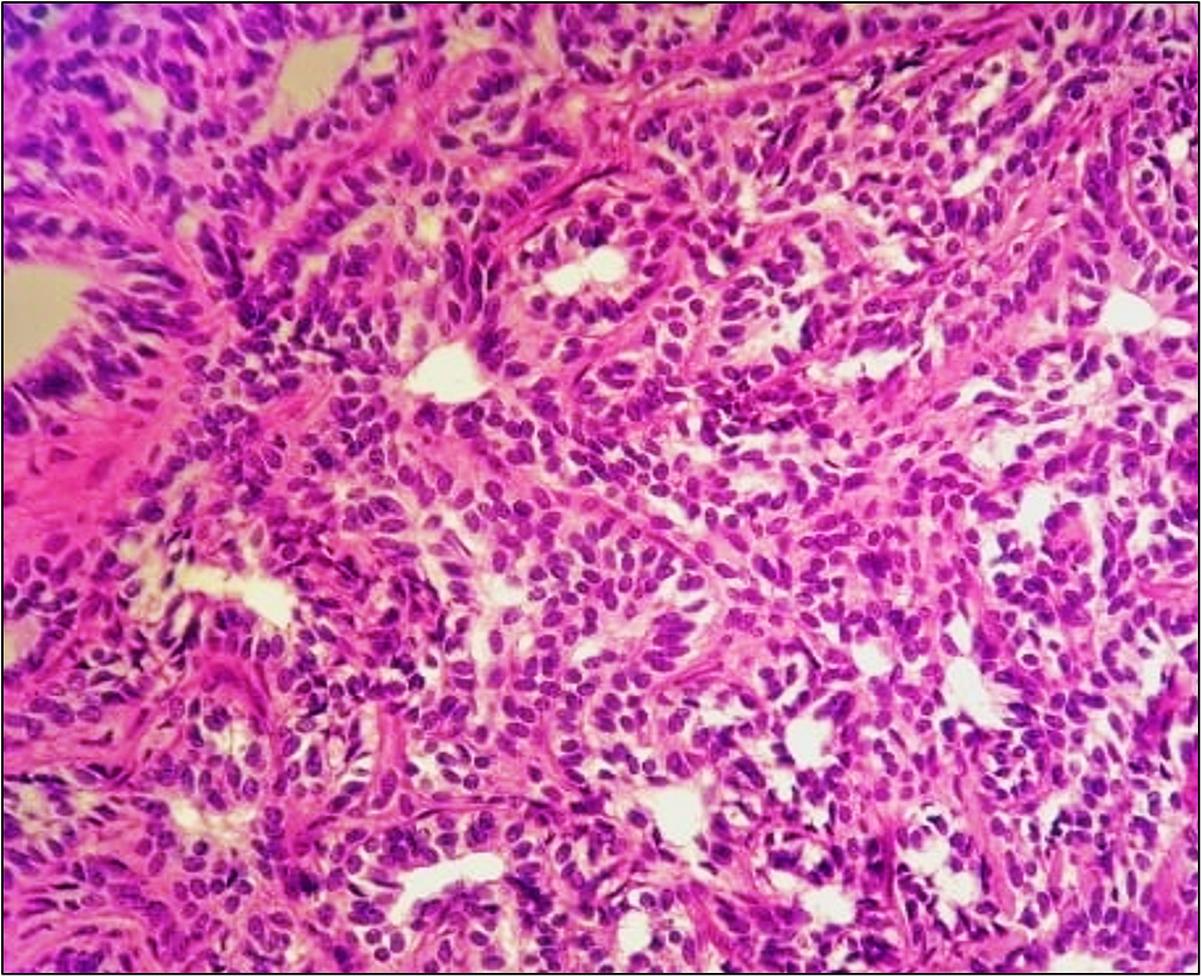
Pathology Image This microscopic section reveals a multi-nodular tumor composed of sheets, nests, cords, and tubules of cells having uniform oval nuclei with a small amount of pale eosinophilic to clear cytoplasm.

Histopathological examination of the resected specimen revealed a multinodular tumor composed of sheets of cells with uniform oval nuclei and pale cytoplasm (Figure 1). Immunohistochemical staining showed positivity for calretinin, neprilysin (CD10), desmin, Wilms tumor 1 (WM1), cytokeratin (CK) AE1/AE3, cluster of differentiation 99 (CD99), and negativity for smooth muscle actin (SMA). In light of these findings, the diagnosis of uterine tumors resembling ovarian sex cord tumors was made.

**Figure 2:**
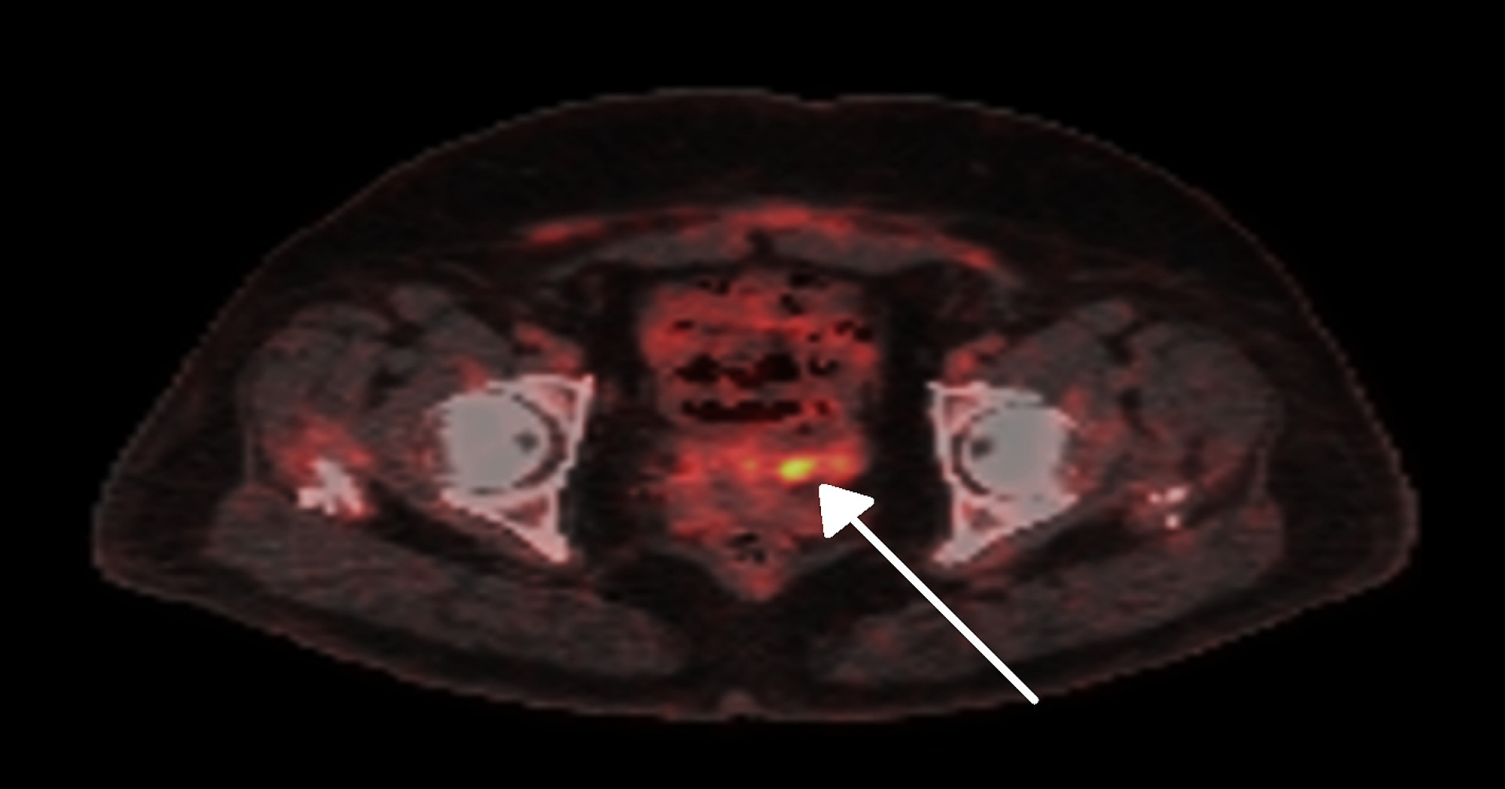
Positron Emission Tomography Image Positron emission tomography image showing an area of irregular increased uptake corresponding to ill-defined hyper-metabolic potentially malignant pelvic mass mostly related to the upper aspect of the vagina (arrow).

A few weeks after the operation, the patient presented with severe lower abdominal pain with no associated symptoms. The pain was not resolved by symptomatic treatment. Abdominal magnetic resonance imaging demonstrated an ill-defined mass, measuring 6.5 cm × 2.6 cm × 2.8 cm, located at the superior part of the vagina with a significant contrast-enhancement. Multiple bilateral pelvic lymphadenopathies were observed. Subsequently, the patient underwent a positron emission tomography scan of an ill-defined hypermetabolic area corresponding to the upper part of the vagina (Figure 2).

The findings were discussed in the multidisciplinary oncology meeting and it was suggested that the patient had a local relapse of the tumor and a surgical resection was planned. The patient underwent complete resection of the pelvic mass with para-aortic lymphadenectomy. Histopathological examination of the lesion revealed the diagnosis of uterine tumor resembling ovarian tumors. However, the lymph nodes showed no evidence of malignancy. The patient was discharged and was symptom-free at the follow-up visits 24 months after the initial presentation.

## Discussion

We described a rare case of uterine tumor resembling a sex-cord tumor in a middle-aged woman with heavy menstrual bleeding. Generally, this tumor displays a benign biological behavior. It usually affects women in the fourth to sixth decade of life [2]. While it typically involves the body of the uterus, the involvement of the endocervix has been reported. It usually manifests as an intramural lesion.

The exact pathogenesis of this tumor remains unclear. Several postulated theories have been suggested to explain the origin of this tumor. For example, the tumor may be originated from undifferentiated mesenchymal stem cells or from ovarian sex-cord cells that have been displaced during embryological development [5]. As in the present case, patients may present with abnormal vaginal bleeding and pelvic pain. Imaging studies demonstrate non-specific findings and the definite diagnosis is reached by histopathological examination [2]. Sadeh et al. [6] suggested that the four immunohistochemistry strains, including calretinin, inhibin, CD99, and Melan A are the most characteristic stains for this tumor. While it usually displays benign behavior, however, our case along with few cases reported in the literature suggests the possibility of recurrence and metastases [1,7,8].

Considering the rarity of the tumor, there are no established treatment guidelines. The management should be discussed in a multidisciplinary meeting by taking the patient’s preferences into consideration [1]. In our case, since the patient completed her family, the decision for a total abdominal hysterectomy was taken. Organ-preserving surgery may be considered in young patients with pregnancy desires [9].

## Conclusions

Uterine tumors resembling sex-cord tumors are a unique group of uterine neoplasms that exhibits diverge clinical and biological characteristic. Surgical pathologists must recognize this rare entity and differentiate it from other lesions. The tumor should be kept in mind in patients with heavy vaginal bleeding in premenopausal and postmenopausal women. Long-term follow-up is essential because these tumors could have recurrence or relapse.
